# Cold tolerance and silencing of three cold-tolerance genes of overwintering Chinese white pine larvae

**DOI:** 10.1038/srep34698

**Published:** 2016-10-05

**Authors:** Juan Wang, Ran-Ran Zhang, Guan-Qun Gao, Ming-Yuan Ma, Hui Chen

**Affiliations:** 1 College of Forestry, Northwest A & F University, Yangling, Shaanxi 712100, China

## Abstract

The values of physiological indices and the enzymes activities involved in the overwintering stage were studied in *D. armandi* larvae in each month from October 2014 to March 2015. The sorbitol, trehalose and glycerol values initially tended to increase as the ambient temperature decreased, before declining until the end of the winter. The activities of four enzymes (SOD, CAT, LDH and AchE) decreased, whereas POD, PK and MDH showed opposite trends in activity. Other enzyme activities (those of TPS, SDH and GLK) were low during the overwintering period and later increased and stabilized during spring. In this study, a polymerase chain reaction (PCR) genes of SDH, TPS and GLK was utilized to identify DarmSDH, DarmTPS and DarmGLK in *D. armandi*. They were found to be abundantly expressed during the overwintering stage by quantitative real-time PCR (qRT-PCR) analyses; by contrast, these three genes showed higher expression levels in December 2014 than in May 2015. The qRT-PCR results demonstrated that the reduction of mRNA expression levels was significant in DarmSDH-, DarmTPS- and DarmGLK-dsRNA-treated *D. armandi* compared with water-injected and non-injected controls. The mortality responses at low temperature were also increased in the dsRNA-treated *D. armandi* compared with the controls.

In temperate regions, insects face a great challenge in surviving at extremely low temperatures^1^,^2^. Overwinter survival is a dominant factor limiting their distribution. Therefore, because of temperature change, higher winter temperature minima every year are regarded as an important determinant of insect range expansion and on a growing in outbreak frequency^3^. An inspect species’ capacity for cold hardiness influences its population dynamics^4^ and geographical distribution^5^. All bark beetle species finish their life cycle under the bark of host trees, except for a short dispersal period when adults find mates and new host trees^6^.

The cold-tolerance of insects is an intricate adaptive response that relates to many defined biochemical, physiological and endocrinological adjustments in insects^7^. In temperate climates, cold tolerance is often closely related to time in overwintering insects, whereas survival primarily depends on physiological and biochemical changes that take place in response to low ambient temperatures^8^. *Dendroctonus armandi* (Tsai and Li) (Coleoptera: Curculionidae: Scolytinae) belongs to a group of insects whose biochemistry and physiology have not yet been fully recognized. Cold tolerance is controlled by more than one environmental condition^9^. Of these conditions, the significance of temperature on the stimulation, maintenance and termination of cold tolerance has been well documented^10^; particularly, low temperature can immediately influence the occurrence, distribution and reproduction of some insects. Additionally, insects have developed various mechanisms that allow them to survive under disadvantageous winter conditions^11^. When insects experience winter, their metabolic activity rate is generally low, and there is no change in their state, organ development or tissue differentiation^12^. However, their physiological metabolic processes continue, such as endocrine regulation, energy metabolism, lipid metabolism and sugar metabolism, among others^13^,^14^. Numerous attempts have been made to illuminate the physiological and biochemical mechanisms related to insect cold-hardiness^11^,^15^,^16^.

Many studies have proved that low-molecular-weight sugars and polyols are significant intermediate metabolites and energy substances in many insect species during the overwintering stage^3^,^4^. Polyols and sugars generally accumulate and act as cryoprotectants under lethally low temperatures^17^. These compounds usually include sorbitol, trehalose and glycerol^18^,^19^. Although different insects accumulate different polyols, the increase in glycerol content is well associated with the strengthening of cold tolerance^11^,^15^,^16^. The metabolic adjustment of these compounds can guarantee efficient resource utilization, maintain the dynamic balance of nutrition, and heighten the level of cold hardiness of overwintering insects, therefore increasing the chances of winter survival^2^. Pullin *et al*.^20^ suggested that the inhibition of some metabolic pathways in advance of low-temperature exposure may avoid the damaging imbalance that may occur when enzyme activities change relative to each other as temperature decreases. Thus, studies of relative enzyme activities and expressions in insects demonstrate that the regulation of equilibrium influences multiple physiological processes and may damage survival, growth, development and insect life span^21^. Certain studies suggested that the biochemical mechanisms of freezing injury and cold hardiness are likely to be connected with antioxidant defence^22^. Oxidative stress and antioxidants, as well as related enzyme activity, are the subject of relatively few studies in cold-hardy insects^8^,^9^. The relative enzyme activity changes in overwintering *D. armandi* are not well understood. To investigate the comprehensive mechanisms of *D. armandi* physiological adaptation to environment changes, its antioxidant enzymes (superoxide dismutase, SOD; catalase, CAT and peroxidase, POD) were determined. In addition, its glycometabolic enzymes (pyruvate kinase, PK; lactate dehydrogenase, LDH; malate dehydrogenase, MDH; sorbitol dehydrogenase, SDH; trehalose-6-phosphate synthase, TPS and glycerol kinase, GLK), esterase (acetylcholinesterase, AchE) activities and the quantitative expression of its genes were measured. We hypothesize that the physiological and biochemical alterations and metabolic adjustments to these changes are connected with changes in the enzyme defence system. The antioxidant defence system, which prevents oxidative injuries from developing during normal metabolic activity, may be significantly altered by cold stress, due to changes characteristic of cold-hardiness mechanisms.

Bark beetles (Coleoptera: Curculionidae: Scolytinae) are endophytic parasites of shrubs and trees and have been broadly recognized for their economic and ecological significance in forests^23^. The Chinese white pine beetle, *D. armandi*, is the most damaging forest insect that invades the phloem of *Pinus armandi* Franch in the Qinling Mountains, Shaanxi, China^24^. Since 1954, *P. armandi* in the Qinling Mountains have been susceptible to ruinous damage caused by approximately 20 species of bark beetles, with *D. armandi*, in particular, a leading serious forest pest, giving rise to large numbers of deaths among otherwise healthy *P. armandi* trees over the age of 30 years^24^. During the process of attacking, females are the first to bore through the bark of the host. The females then attract males with sex pheromones for colonization and reproduction^6^ and oviposit under the bark of the host trees. The larvae live through the winter, after which the next generation of adults emerges. This indigenous insect pest causes extensive tree mortality in natural forest ecosystems, reaching epidemic proportions and similar to most insects, its cold-season survival depends mainly on cryoprotectants.

Trehalose (α-D-glucopyranosyl-1,1-α-D-glucopyranoside) is an important disaccharide^25^,^26^, is widespread among bacteria, yeasts, fungi, nematodes, plants, insects and some other invertebrates^27^. The accumulation of trehalose is involved in adaptations to adverse environmental stresses that include dehydration, heat, freezing, desiccation and oxidation^28^. Trehalose always serves as a stabilizing agent for cellular structures under stress conditions and has a special capacity for the protection of cellular membranes and proteins from the undesirable effects of heat, cold and dehydration^27^,^29^. During the overwintering stage, cryoprotective compounds, such as trehalose, sorbitol and glycerol, accumulate to enhance survival^18^,^19^. Trehalose is the main blood sugar in insects, which synthesize it in the fat body before its release into the haemolymph^30^,^31^,^32^,^33^. This sugar is thought to be necessary for thermotolerance in larvae and is involved in regulating pupal diapause^30^,^32^,^34^. Moreover, it is also considered an important factor in coping with environmental stress, especially low-temperature stress. Therefore, trehalose and its metabolism are pivotal for the growth, development and survival of insects^34^. The biosynthesis of trehalose is known: trehalose-6-phosphate synthase (TPS: E.C.2.4.1.15), catalyses the reaction of uridine diphosphate glucose (UDPG) and glucose-6-phosphate to form trehalose-6-phosphate, which is converted into free trehalose by trehalose-6-phosphate phosphatase (TPP: E.C.3.1.3.12). As trehalose is the main reserve sugar in the haemolymph, serving as a carbon and energy source for flying insects^33^, it is meaningful to study the function of TPS, a rate-limiting enzyme in trehalose synthesis^34^ and one of the main factors regulating trehalose levels in the insect haemolymph.

SDH is a member of the zinc-dependent alcohol dehydrogenase-like (ADH) family^35^. The process by which SDH catalyzes the interconversion of polyols and ketoses begins when the zinc atom associates with three amino acid residues and a water molecule; this process requires NAD^+^ as a cofactor^36^. To acclimate to cold weather, insect overwintering eggs often contain sorbitol, in addition to glycerol^36^. SDH activity is not induced in overwintering eggs of *Bombyx* exposed to temperature of 0 °C^37^, and therefore sorbitol is not utilized. The accumulation of sorbitol, resulting from an overload of glucose from glycogen^38^, was also be examined in the resting eggs of flesh flies^39^. Therefore, the storage of sorbitol is a direct response to extremely low temperatures^40^. Sorbitol accumulation depends on the activity of SDH, which was not induced in the overwintering eggs. In overwintering *Bombyx* eggs, SDH is normally expressed at a very low level. However, when the eggs are transferred from approximately 0 °C to 25 °C, SDH activity strongly increases^37^; this increase with warming also occurs for the diapause eggs of *Drosophila*^41^.

Glycerol can be biosynthesized via two biosynthetic pathways via glycolysis and the pentose phosphate cycle^42^. One pathway forms glycerol from glyceraldehyde-3-phosphate (GAP)^43^,^44^. GAP is dephosphorylated by glyceraldehyde-3-phosphatase to produce glyceraldehyde, which is then reduced by polyol dehydrogenase (PDH), with a reducing power of NADPH + H^+^. as seen in *Epiblema scudderiana* (Clemens)^45^. Another pathway for glycerol formation is that from DHAP via GPDH and glycerol-3-phosphatase or GLK, as seen in *Spodoptera exigua*^46^. GLK catalyzes glycerol phosphorylation for the utilization of glycerol^47^,^48^. In overwintering *Hyalophora cecropia* eggs, which accumulate glycerol, GLK plays a role in overwintering termination by converting glycerol to glycerol-3-phosphate for other intermediary metabolism^49^. Park and Kim^46^ suggested that GLK catalyzes the reverse reaction of dephosphorylation of glycerol-3-phosphate to produce glycerol because RNA interference (RNAi) of GLK significantly reduced glycerol accumulation in a 4 °C pretreatment. In *Bombyx mori*, at least three GLK isozymes have been reported, among which only one isoform, GK3, appears to be associated with the utilization of glycerol^50^.

RNAi is a mechanism for post-transcriptional gene silencing that has been developed as a powerful tool for studying gene function in a variety of organisms^51^. Since RNAi was first discovered in the nematode *Caenorhabditis elegans*^52^, scientists have explored a variety of efficient means to transport double-stranded RNA (dsRNA) into organisms, including microinjection, soaking and oral feeding^53^,^54^. Two research groups, in particular, have made prodigious progress in exploiting transgenic plants that express insect dsRNAs for entomological research and insect pest control, suggesting that RNAi could be a useful pest control method. To achieve this goal, large-scale screening methods for pest gene pools are required^55^. For small insects, such as *D. armandi*, microinjection is considered an excellent method because of its convenience and low rate of injury. By silencing different genes, the roles of diverse proteins have been researched under environmental stress, especially at low temperature^56^,^57^.

The main objectives of the current study were to assess cold tolerance and to determine the means by which the physiological index values and enzyme activities, as well as the DarmSDH (sorbitol dehydrogenase, SDH), DarmTPS (trehalose-6-phosphate synthase, TPS) and DarmGLK (glycerol kinase, GLK), affect *D. armandi* larvae during overwintering. Determining how Chinese white pine beetles respond to the physiological problems posed by winter conditions is vital for predicting epidemic situations and their long-term impact. Therefore, the study of these problems in *D. armandi* has an important role in understanding the coordinated effects on the overwintering larval body.

## Results

### Physiological indices

**Figure 1:**
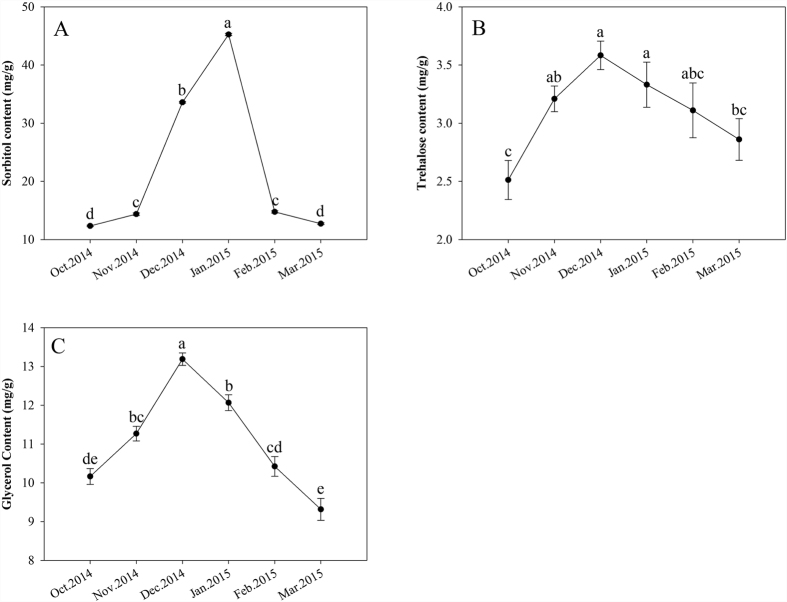
Content changes of sorbitol, trehalose and glycerol in the Chinese white pine beetle (*Dendroctonus armandi*) larvae at six sampling time points from October 2014 to March 2015. Note: Values are presented as means ± SE. Values with the different lowercase letters in the contents are significantly different (P < 0.05, Tukey’s multiple comparisons test after analysis of variance).

Three major physiological indices were examined in the overwintering larvae, and all three indices varied significantly throughout the overall overwintering period (Fig. 1). The sorbitol content started at a low level of 12.35 ± 0.12 mg/g in October and quickly climbed to a level of 33.60 ± 0.13 mg/g and 45.26 ± 0.2 mg/g in December and January, respectively (*F* = 2.163 × 10^4^, df = 5, P < 0.001), then declined to a level of 12.72 ± 0.16 mg/g in March (Fig. 1A). Additionally, the trehalose contents clearly increased during October to December, reaching a peak at 3.58 ± 0.12 mg/g in December during the middle of the overwintering period (*F* = 9.378, df = 5, P < 0.001), and then steadily declined to a value of 2.86 ± 0.18 mg/g in March (Fig. 1B). The glycerol content increased from a low level of 10.16 ± 0.20 mg/g in October to its highest level of 13.19 ± 0.16 mg/g in December (*F* = 5.048 × 10, df = 5, P < 0.001) and then declined again to a level of 9.32 ± 0.28 mg/g in March (Fig. 1C).

### Enzyme activity assays

**Figure 2:**
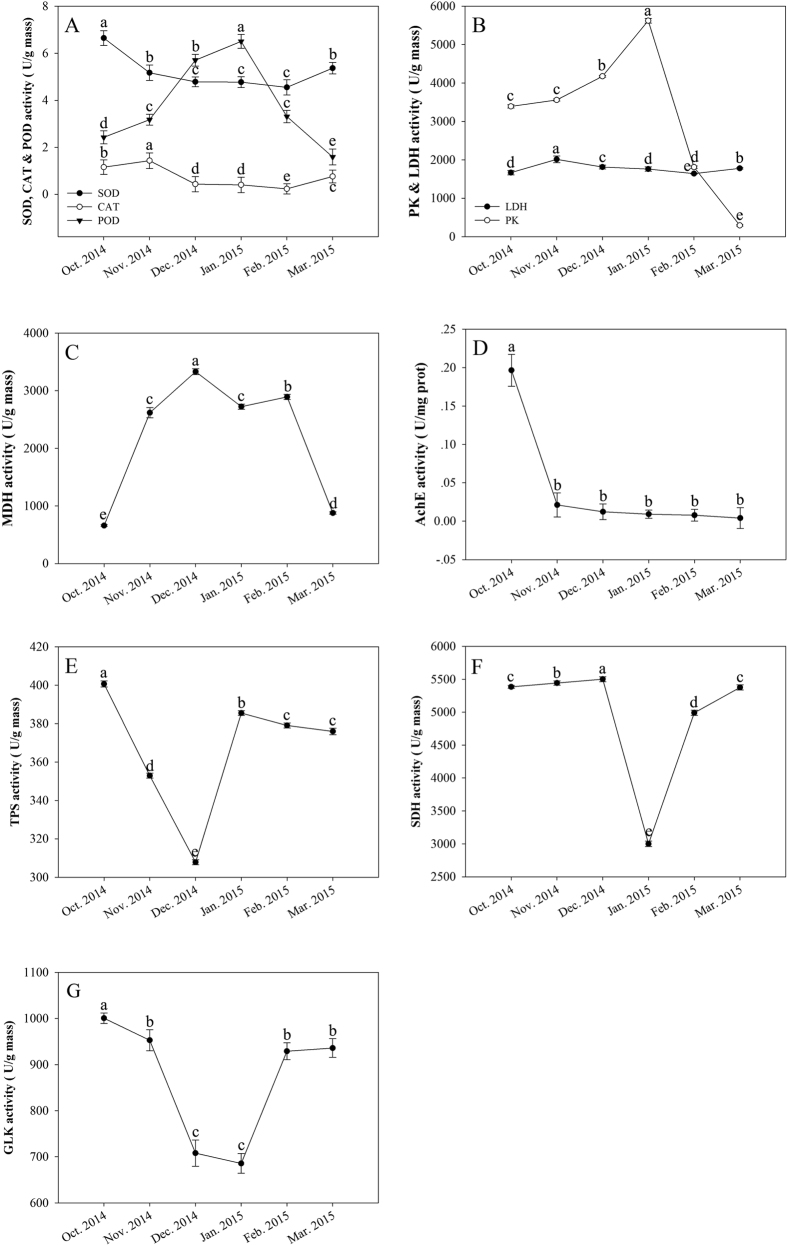
Changes of enzyme activities (including SOD,CAT, POD, PK, LDH, MDH, AchE, TPS, SDH and GLK) in Chinese white pine beetle (*Dendroctonus armandi*) larvae in each month from October 2014 to March 2015. Note: Values are presented as means ± SE. Values with the different letters are significantly different (p < 0.05) (Tukey’s multiple comparisons test after analysis of variance). Abbreviation: SOD: superoxide dismutase; CAT: catalase; POD: peroxidase; PK: pyruvate kinase; LDH: lactic dehydrogenase; MDH: malate dehydrogenase; AchE: acetylcholine esterase; TPS: trehalose-6-phosphate synthase; SDH: sorbitol dehydrogenase; GLK: glycerol kinase.

The SOD and CAT activities changed little during each month on six sampling times and slightly decreased from November to February. The SOD (*F* = 1.945 × 10^2^, df = 5, P < 0.001) and CAT (*F* = 2.007 × 10^2^, df = 5, P < 0.001) activities consistently decreased at a very low rate in October, then decreased slightly during the overwintering period (between November and February), and weakly increased in March (Fig. 2A). However, the POD (*F* = 1.934 × 10^2^, df = 5, P < 0.001) activity changed inversely with the activities of the first two enzymes. Compared with the SOD and CAT activities, the POD activity changed significantly in each month during the winter. The POD activity increased from October to January, when the maximum POD activity was 6.503 ± 0.3 (g mass)^−1^, almost three times the value recorded in October (beginning of the overwintering stage). After reaching a maximum, the POD activity sharply decreased to the March level (Fig. 2A).

The PK activity significantly increased under low temperatures from October to January. In January, the PK (*F* = 1.041 × 10^7^, df = 5, P < 0.001) activity reached its highest level of 5622.66 ± 60.34 (g mass)^−1^ from a starting point of 3390.43 ± 41.70 (g mass)^−1^ in October. In February, the PK activity significantly decreased to 1808.79 ± 31.02 (g mass)^−1^ and reached its lowest level of 292.86 ± 17.28 (g mass)^−1^ in March (Fig. 2B). The LDH (*F* = 9.71 × 10^5^, df = 5, P < 0.001) activity changed insignificantly in each month on six sampling times, only increasing gradually in November to a level of 2010.28 ± 87.76 (g mass)^−1^ and then decreasing slowly to 1777.11 ± 14.84 (g mass)^−1^ in March (Fig. 2B).

The MDH activity changed sharply during the winter and was significantly higher (*F* = 3.80 × 10^6^, df = 5, P < 0.001) in December compared with October and November. The MDH activity reached a level of 3331.02 ± 50.91 (g mass)^−1^ in December, declined to 2723.59 ± 44.91 (g mass)^−1^ in January and then recovered slightly. Finally, the level of MDH activity fell to 876.25 ± 20.83 (g mass)^−1^, its lowest value, in February (Fig. 2C).

Information on the AchE activity is given in Fig. 2D. The AchE (*F* = 6.220 × 10, df = 5, P < 0.001) activity was 0.20 ± 0.02 (mg prot)^−1^ in October. With decreasing environmental temperature, the AchE activity declined quickly to 0.02 ± 0.01 (mg prot)^−1^ in November. After that, the AchE activity dropped further to reach a minimum value of 4.081 × 10^–3^ ± 0.01 (mg prot)^−1^ in March 2015.

Information on TPS, SDH and GLK activities is shown in Fig. 2E–G. The tendency was for TPS (*F* = 4.267 × 10^2^, df = 5, P < 0.001) activity to decrease significantly from 400.66 ± 1.63 (g mass)^−1^ in October to 306.79 ± 1.33 (g mass)^−1^ in Deember when it reached its minimum level. Then, the TPS activity increased sharply in January to 385.39 ± 1.24 (g mass)^−1^. After that, the TPS activity fluctuated within a narrow range (Fig. 2E). The activity of SDH showed the same decreasing trend as that of TPS, although it occurred during different winter months. The activity rose slightly from October to December and reached a value of 5501.37 ± 38.31 (g mass)^−1^. Then, the SDH activity (*F* = 2.83 × 10^6^, df = 5, P < 0.001) dropped sharply to 2999.59 ± 41.40 (g mass)^−1^ in January. After that, the activity kept rising into March to a value of 5376.54 ± 40.30 (g mass)^−1^, recovering the same level as that before overwintering (Fig. 2F). The GLK activity (*F* = 7.460 × 10^2^, df = 5, P < 0.001) declined during the winter to reach a minimal level in January of 685.51 ± 21.40 (g mass)^−1^. The GLK activity levels remained high before December and stable between February (929.03 ± 18.26 (g mass)^−1^) and March (935.95 ± 20.30 (g mass)^−1^). The levels decreased from 1000.66 ± 11.40 (g mass)^−1^ in October to 707.82 ± 28.31 (g mass)^−1^ in December (Fig. 2G).

### Mortality at low temperatures and LT_50_

**Figure 3:**
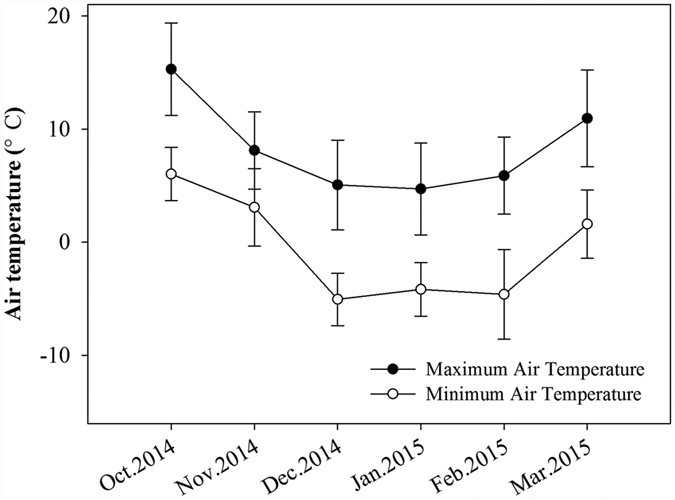
Minimum and maximum air temperature (monthly mean ± SE) from October 2014 to March 2015. Note: The data was provided by the Huoditang, Qingling Forest Protection Station, Northwest A&F University, Shaanxi, China (The sampling area locates on the southern slope of the middle Qinling Mountains (33°18′–33°28′N, 108°21′–108°39′E), Shaanxi, China).

**Figure 4:**
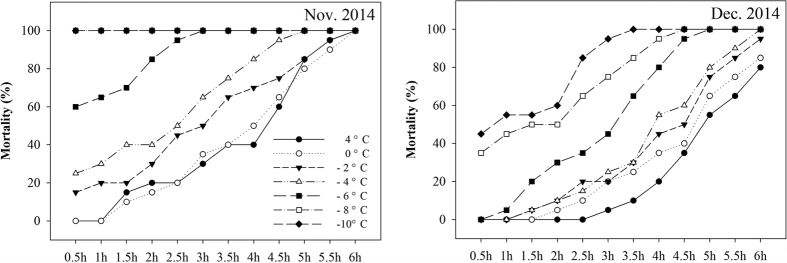
Mortality of Chinese white pine beetle (*Dendroctonus armandi*) larvae at different low temperature (4, 0, −2, −4, −6, −8 and −10 °C) for different times (0.5, 1, 1.5, 2, 2.5, 3, 3.5, 4, 4.5, 5, 5.5 and 6 hours) in November and December 2014.

The ability of *D. armandi* larvae to survive at low temperatures improved constantly as the mean monthly environmental temperature decreased from 5.0 ± 3.4 °C in November to 0.0 ± 3.1 °C in December (Fig. 3). Larval mortality was detected in *D. armandi* during November and December 2014 at low temperatures (Fig. 4). In November, the larvae could tolerate temperatures of −6 °C for 0.5 h, but the mortality was 60%. The mortality increased to 100% when the larvae were held at −6 °C for 3 h. By December, the mortality was 0% at −6 °C for 0.5 h. The larvae could tolerate temperatures of −6 °C for 5 h, but the mortality was up to 100%. Furthermore, the larvae could tolerate a low temperature of −8 °C and −10 °C for 4.5 h in December but could not do so in November (when mortality was 100% after 0.5 h).

The LT_50_ values of overwintering larvae in November 2014 for each exposure time (0.5, 1, 1.5, 2, 2.5, 3, 3.5, 4, 4.5, 5, 5.5 and 6 h) were −5.7,−5.4, −4.8, −4.4, −4, −2, −0.9, 0, 4.4, 4.7, 4.9 and 5.4 °C, respectively. However, the LT_50_ values in December 2014 were −10.4, −9.0, −8.0, −8.0, −7.8, −6.5, −5.9, −3.7, −2, 4.1, 4.3 and 5 °C, respectively (Table 1). As seen from these results, low-temperature resistance was stronger in December than that in November. The higher cold tolerance of larvae was confirmed by their greater ability to survive exposures to low temperature.

### cDNA sequence analyses of DarmSDH, DarmTPS and DarmGLK

**Figure 5:**
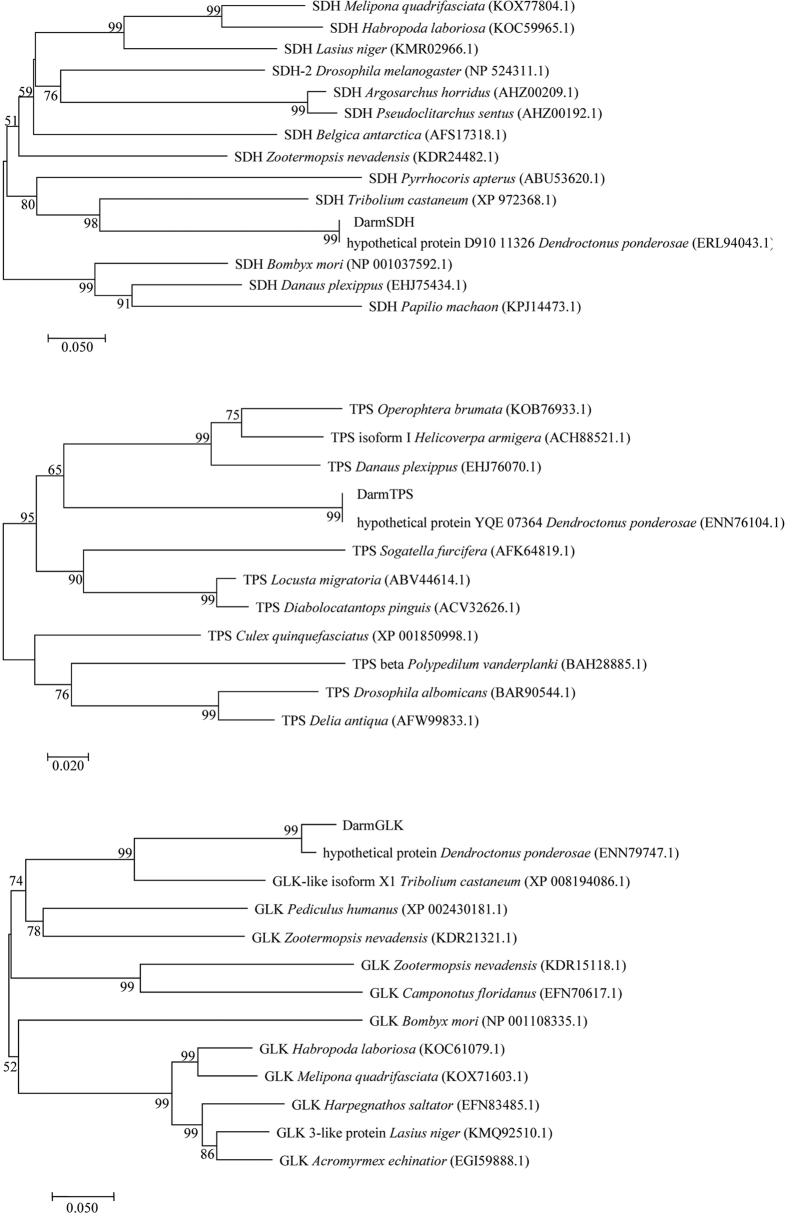
Phylogenetic analysis of DarmSDH, DarmTPS and DarmGLK genes from *Dendroctonus armandi* and SDH, TPS and GLK from other insects constructed by the neighbor-joining method based on amino acid sequences. The accession numbers of outgroup GenBank sequences are shown in parentheses. Percentage bootstrap values above 50% were indicated on each cluster, and values below 50% were omitted.

We successfully obtained the DarmSDH, DarmTPS and DarmGLK cDNA fragments (735 bp, 1199 bp and 1033 bp, respectively) by PCR using specific primers (Table 2). BLAST searches indicated that these three genes expressed in *D. armandi* were similar to the genes reported in other insect species (Table 3). Identity between each gene and the GenBank reference sequences was 51–99%. Additionally, three phylogenetic trees were constructed by using protein sequences from different insect genera and families (Fig. 5). The insects represented on the phylogenetic trees include Coleoptera, Hymenoptera, Hemiptera, Lepidoptera, Orthoptera and Diptera. As expected, *D. armandi* DarmSDH, DarmTPS and DarmGLK are rooted in the Coleoptera group with *Dendroctonus ponderosae* and *Tribolium castaneum*. Although, there were no names for the protein sequences of *D. ponderosae*, we could also find the results among the other insect protein sequences.

### Expressions of DarmSDH, DarmTPS and DarmGLK

**Figure 6:**
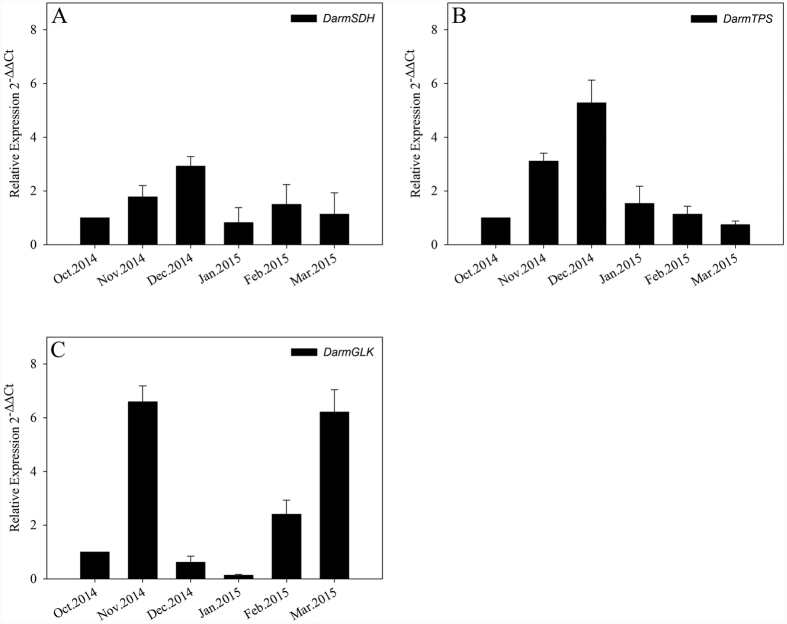
Relative mRNA expression levels of the DarmSDH (**A**) DarmTPS (**B**) and DarmGLK (**C**) in each month from October 2014 to March 2015. The relative expression levels were normalized by actin, with the expression in October 2014 as the calibrator. The standard errors of the means of three biological replicates are represented by error bars.

Real-time PCR analysis was used to detect the transcript levels of DarmSDH, DarmTPS and DarmGLK in *D. armandi* larvae during different months from October 2014 to March 2015 (Fig. 6). The level of DarmSDH was up-regulated during the overwintering stage (Fig. 6A). Significant up-regulation of DarmTPS was observed with the arrival of winter; DarmTPS expression reached its maximum level in December (Fig. 6B). By contrast, the DarmGLK expression level decreased notably from November to January, reaching an extremely low level in January, but increased from February through March to nearly to the same level that was observed in November (Fig. 6C).

**Figure 7:**
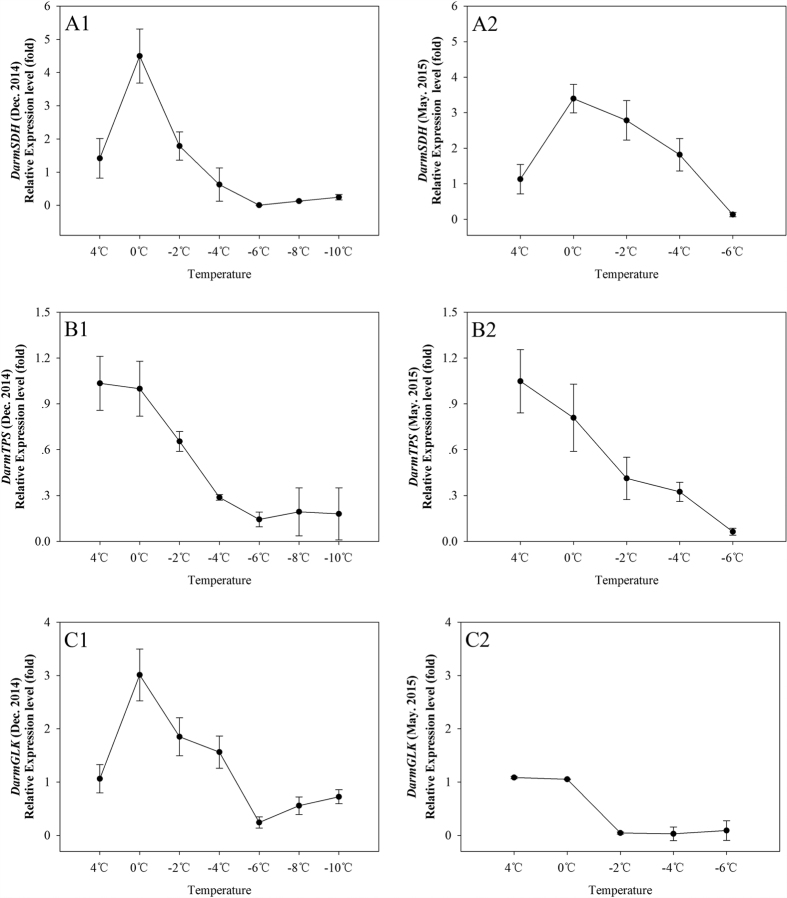
Relative mRNA expression levels of the DarmSDH (A1, A2), DarmTPS (B1, B2) and DarmGLK (C1, C2) in December 2014 and May 2015 at different low temperatures (Dec. 2014 for 4, 0, −2, −4, −6, −8 and −10 °C May 2015 for 4, 0, −2, −4 and −6 °C) for 12 h. The relative expression levels were normalized by actin, with the expression at 4 °C as the calibrator. The standard errors of the means of three biological replicates are represented by error bars.

The relative mRNA levels of DarmSDH, DarmTPS and DarmGLK genes were quantified by real-time quantitative PCR in December 2014 and May 2015 for comparison. We found the expression levels of these three genes changed irregularly with time of day over 24 h (not shown). Finally, we chose to induce their expression by a low-temperature exposure for 12 h (Fig. 7). The expression levels of DarmSDH in December 2014 showed up-regulated expression with low temperature variation. For the 0 °C treatment in May 2015, the expression levels of DarmSDH appeared lower than those observed in December 2014, but the levels still exceeded those recorded in calibrators (Fig. 6A1,A2). The larval expression of DarmTPS in December 2014 showed the same down-regulated trends as were shown in May 2015 for different low temperatures (Fig. 6B1,B2). The DarmGLK expression was up-regulated from 0 °C to −4 °C in December 2014, whereas the transcriptional expressions in May 2015 were remarkably decreased with different low temperatures (Fig. 7C1,C2).

### Effect of dsRNA treatments on DarmSDH, DarmTPS and DarmGLK

**Figure 8:**
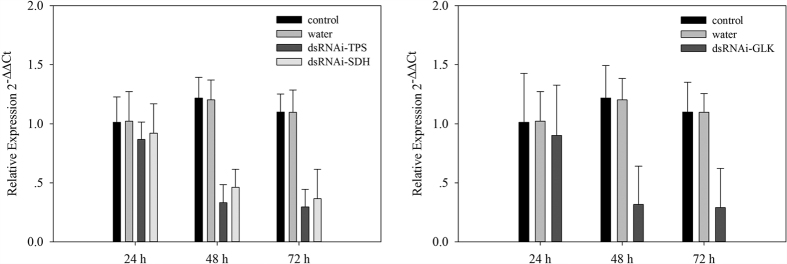
qRT-PCR analysis of DarmSDH, DarmTPS and DarmGLK transcript patterns from *D. armandi* larvae; after injected for 24 h, 48 h and 72 h. The standard errors of the means of three biological replicates are represented by error bars. Transcript patterns of DarmSDH and DarmTPS were analyzed on December 2014, and DarmGLK on November 2014.

The transcriptional expression level of DarmGLK reached its maximum value in November, while DarmSDH and DarmTPS reached their peak values in December (Fig. 6). We knocked down the DarmSDH, DarmTPS (December 2014) and DarmGLK (November 2014) genes by RNAi to study the influence of the knockdown on mortality. There were significant differences among the non-injected, water-injected and dsRNA-injected groups 24, 48 and 72 h after dsRNA injection (Table 4). The results in Fig. 8 showed that the expression level of dsRNAi-SDH dropped approximately 2.5 times, whereas that of dsRNAi-TPS and dsRNAi-GLK was reduced about three times at 72 h compared with 24 h after injection. Furthermore, the transcript levels of DarmSDH, DarmTPS and DarmGLK in the non-injected and water-injected *D. armandi* larvae remained unchanged.

### Effect of dsRNA treatments on mortality responses to low temperatures

**Figure 9:**
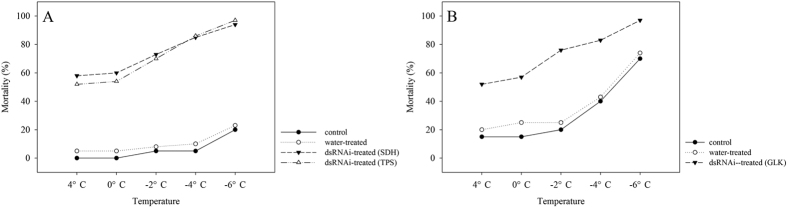
Mortality responses of dsRNAi-treated, water-injected, and non-injected *D. armandi* larvae to different low temperature (4, 0, −2, −4 and −6 °C) for 1.5 h. Larvae mortality of DarmSDH and DarmTPS was measured on December 2014, and DarmGLK on November 2014.

We examined the responses of dsRNA-injected, water-injected, and non-injected *D. armandi* larvae to low temperature by mortality analysis. (The larvae were collected in December 2014 for dsRNAi-SDH and dsRNAi-TPS, and in November 2014 for dsRNAi-GLK). The mortality changed significantly with different durations of low-temperature exposure (Fig. 4). We chose the duration of 1.5 h for an exposure between 4 °C and −6 °C to detect the effect of dsRNA treatment while minimizing the impact of external factors. After low-temperature exposure, the mortality of the dsRNA-treated larvae was remarkably higher than that of the water-injected and non-injected controls (P < 0.0001) (Fig. 9). At 4 °C, the mortality of the dsRNA-treated larvae was approximately six times than that of water-injected and non-injected controls for dsRNAi-SDH and dsRNAi-TPS An obvious increase in mortality occurred when the larvae were injected with dsRNAi-SDH, dsRNAi-TPS and dsRNAi-GLK (Fig. 9).

## Discussion

Cold hardiness is an essential component of winter survival for most insects in temperate zones^2^,^4^,^5^. Cold hardiness provides tolerance to low temperatures and allows overwintering insects to sustain crucial bodily functions in unfavourable environments without feeding^46^. Cold tolerance can be obtained by freeze tolerance, through resistance to internal ice formation^46^. However, large numbers of terrestrial insects are freeze susceptible and thus avoid the formation of internal ice by heightening their supercooling capacity through an increased production of polyols or other cryoprotectants^58^. In the current study, major overwintering physiological indices, enzyme activities, mortalites at low temperatures, overwintering genes’ transcriptional levels and the effects of RNA interference experiments were measured to assess cold tolerance and to determine the mechanisms by which DarmSDH, DarmTPS and DarmGLK function in winter in *D. armandi* larvae. Determining how Chinese white pine beetles respond to low-temperature problems under winter conditions is vital for predicting epidemic situations and their long-term impact.

In overwintering larvae of the Chinese white pine beetle, the sorbitol, trehalose and glycerol contents increased and reached their highest levels during the colder month of the whole year. This finding suggests that overwintering *D. armandi* larvae accumulated low-molecular-weight carbohydrates under field conditions. Most overwintering insects can accumulate low-molecular-weight sugars and polyols as important cryoprotectants^59^. Moreover, glycerol is common in all types of insects^1^,^7^,^60^. In addition, the beetles *Phyllodecta laticollis*^61^ and *Xylotrechus rusticus*^59^ increase their cold hardiness from the summer to the winter by accumulating high concentrations of glycerol. By contrast, a more detailed study noted that sorbitol was produced in great quantities when *Eurosta solidaginis* larvae were exposed to strong cold stress until the body fluids froze^62^. Furthermore, codling moth larvae accumulated high levels of trehalose during the overwintering stage^3^, and this species showed a significant relationship between trehalose content and the development of cold tolerance^3^. The winter accumulation of low-molecular-weight sugars and/or polyols has been well proven in many overwintering insects in temperate regions^2^,^63^,^64^.

Overall, the activities of many enzymes in *D. armandi* larvae changed substantially during the winter season. Some of the observed changes correlated with the synthesis and/or the degradation of polyol cryoprotectants. Others appeared to be related because significant changes in their enzyme activities occurred in overwintering larvae. Clearly, metabolic reorganizations take place throughout the winter in the larvae, with several different patterns distinguished. In the fall, the activities of many enzymes associated with cryoprotectant synthesis increased significantly. During the winter, from November to January in this study, relatively few changes were detected; however, certain enzymatic activities did change during this interval, reflecting the dynamic nature of metabolism even in dormant larvae at low ambient temperatures. Between January and March, numerous changes in enzyme activities appeared to be preparatory for spring warming and the resumption of development. Thus, the activities of enzymes associated with polyol catabolism increased to allow cryoprotectant catabolism as ambient temperatures warmed. Taken together, these results show a complex and flexible system of enzymatic rearrangement in this species in response to acclimatization or developmental needs. The health status and the maintenance of homeostasis in individual organisms are conditioned by the proper state of the antioxidant system, which allows the successful scavenging of free radicals and the reconstruction of damages important for life molecules^15^,^16^,^17^. Low temperatures and a slowing of metabolism do not provide complete protection against oxidative damage, which is why an efficient antioxidant system is crucial for the survival of overwintering insects^65^. Chinese white pine beetles normally overwinter in the phloem of Chinese white pines as larvae. During winter, high levels of cryoprotectants (sorbitol, trehalose and glycerol) accumulated in overwintering larvae, whereas the POD activities increased. These results showed that antioxidant enzymes and carbohydrates share a similar role in the regulation of energy metabolism. Oxygen consumption in aerobic cells is accompanied by the generation of reactive oxygen species (ROS), such as hydrogen peroxide (H_2_O_2_), lipid peroxides, and hydroxyl radicals. If not eliminated, ROS propagate further oxidative processes, leading to the damage of cellular molecules and resulting in disturbed homeostasis and cellular death^66^. The elimination of H_2_O_2_ in insects can be achieved in two ways: (1) by CAT and glutathione recycling and (2) by an ascorbate recycling antioxidant mechanism^66^. We infer that if H_2_O_2_ elimination by CAT and the glutathione recycling mechanism is well balanced and sufficient to prevent oxidative damage, the second mechanism could be less active, and vice versa. This situation points to a precisely coordinated regulation in the levels of ROS and redox-active molecules by antioxidant enzymes. Furthermore, during the overwintering stage, glycogen was converted into other substances^67^, while the activity of SOD and CAT were gradually decreased, which enhanced the protective ability to regulate the cold resistance of overwintering larvae.

Insects are poikilothermic animals, and their cold-tolerance capacity is a key aspect of their adaptation to their geographical environment, including factors such as altitude, latitude, and ambient temperature. Therefore, the determination of mortality at low temperature can help us understand the principles of overwintering in *D. armandi* larvae from a new perspective. The LT_50_ values were low in November and decreased further in December. A possible explanation for this LT_50_ drop might be that low temperature was a limiting factor for larval survival during the overwintering stage. In addition, Boardman *et al*.^68^ demonstrated that fasting larvae of *Thaumatotibia leucotreta* (Meyrick) are more tolerant than those that are fed, using another way of stating the case. Langford^69^ reported that the potato tuber moth survived temperatures ranging from −6.6 to −11.6 °C, but prolonged exposure to these temperatures is apparently lethal for all development stages. Our findings indicated that the LT_50_ for *D. armandi* overwintering larvae declined during winter months to reduce mortality.

The transcript levels in the larvae of the Chinese white pine beetle reached their highest levels for DarmSDH and DarmTPS in December 2014 and for DarmGLK in November 2014; therefore, we chose November and December for the measurement of mortality. The low-temperature-mortality rate increased with longer exposure times and with decreasing temperatures. Additionally, under natural conditions, the cold-tolerance capacity of *D. armandi* larvae in the overwintering stage increased significantly in December compared with November. The effects of low temperature on mortality have also been demonstrated in other insects, such as *Monochamus alternatus*^5^, *Sarcophaga bullata*^70^ and *Drosophila melanogaster*^71^,^72^.

In addition, we quantified changes in the DarmSDH, DarmTPS and DarmGLK concentrations in each month from October 2014 to March 2015 and detected the quantitative expression of these three genes under different low-temperature conditions in May 2015 and December 2014. Sorbitol dehydrogenase is a widely distributed enzyme that, together with aldose reductase, constitutes the sorbitol pathway^73^. Although SDH activity has been detected in a number of mammalian tissues, such as liver, kidney, lens, erythrocytes and the male reproductive system^74^, little is known about the physiological function of SDH of *D. armandi* larvae. In the insect, *Bombyx mori*, SDH has been shown to control the utilization of sorbitol in overwintering eggs^37^. Real-time PCR of overwintering larvae from different months showed that DarmSDH was expressed at relatively high levels in December. In January, DarmSDH was expressed at low levels. The range of expression of DarmSDH was wider in December 2014 than in May 2015, indicating that this protein is crucial for the overwintering process, as it is needed to digest the winter-accumulated sorbitol.

Trehalose represents the primary haemolymph sugar in many insects, and it functions in energy metabolism and for protection under cold environmental conditions^2^,^75^. In insects, the TPS gene was first cloned from *D. melanogaster*^75^,^76^. This gene plays important roles in insect development and tolerance to various stresses^76^,^77^. These findings demonstrate that interfering with trehalose biosynthesis could act as an insecticidal mechanism and that the trehalose biosynthetic enzyme TPS is a latent drug target^75^. TPS is a rate-limiting enzyme in trehalose synthesis^2^,^56^,^75^. Although insect TPS genes have been found in several insects, including *D. melanogaster*^37^, *Helicoverpa armigera*^34^, *Spodoptera exigua*^78^, and *Catantops pinguis*^79^, no TPS gene had been reported in the *D. armandi* larvae. Therefore, the characterization of the DarmTPS gene and its enzyme activity in overwintering larvae was of interest. In our study, the expression pattern of the DarmTPS gene was observed in summer-sampled larvae (May 2015), in overwintering (December 2014) larvae and in larvae collected from October 2014 to March 2015. Nevertheless, significantly greater expression of DarmTPS was observed during winter in the overwintering larvae (Fig. 5B). Considerable research has been conducted to investigate insect TPS genes^34^,^78^,^79^,^80^. In *H. armigera*, the abundance of the HarTPS gene was higher in overwintering pupae than in non-overwintering pupae^34^. In *D. melanogaster*, a mutation in the TPS gene was lethal to young larvae, indicating that it played a critical role in insect development^76^. In *S. exigua*, TPS RNAi also resulted in larval death^78^. We hypothesize that our DarmTPS is an overwintering-response gene in the Chinese white pine beetle that might play a role in adverse-cold-stress resistance. Further work is needed to explore the function of TPS.

The biosynthesis of glycerol follows two independent pathways depending on the insect. One pathway uses polyol dehydrogenase catalysing glyceraldehyde with NADPH + H^+^ (*Epiblema scudderiana*)^81^. The other pathway uses GPDH/GLK from dihydroxyacetone-3-phosphate to glycerol (*S. exigua*)^46^. Although the GLK gene has been detected in a number of insects, such as *E. scudderiana*^81^ and *S. exigua*^46^, little is known about DarmGLK in *D. armandi* larvae. The real-time PCR of overwintering larvae from different months showed that DarmGLK was expressed at relatively high levels in November but at low levels in December and January. Even so, the expression of DarmGLK was higher in December 2014 than it was in May 2015. This indicates that this protein is crucial for the overwintering process.

A variety of efficient methods for the delivery of dsRNA into insects has been explored in recent years to knock down specific gene expression. Owing to its effectiveness, microinjection remains the most direct and popular method of knocking down the expression of target genes^56^. Previous studies have proven that injection-based RNAi can definitely lead to gene silencing in *D. armandi*^82^. The successful knockdown of the DarmSDH, DarmTPS and DarmGLK genes at the mRNA expression level was confirmed in our RNAi experimental system. The injection of dsRNA (0.1 μM per larva) of the olfactory co-receptor gene in *D. armandi* reduced the expression of the target gene by 80%^82^. Therefore, the hypothesis that RNAi can be used as a functional genomic tool in the Coleopteran species *D. armandi* can now be considered a tenable hypothesis. We have demonstrated the feasibility of injecting RNAi and shown its injection can demonstrably inhibit the transcription level of a target gene in *D. armandi* larvae. This effect was also observed after abdominal injections of dsRNA: the midgut aminopeptidase N gene in *Spodoptera litura*^83^ and the vitellogenin gene in adult honeybees^84^ were almost totally silenced. The knockdown of the target genes DarmSDH, DarmTPS and DarmGLK not only suppresses their transcription levels but also affects larval cold-tolerance capacity, leading to an increasing mortality rate at low temperature. The qRT-PCR results of the RNAi experiments demonstrated that the levels of mRNA expression in the *D. armandi* larvae treated with DarmSDH, DarmTPS and DarmGLK dsRNA were significantly reduced compared with the levels in the two controls (Fig. 7). We examined the mortality of dsRNA-injected, water-injected, and non-injected *D. armandi* larvae exposed to low temperatures (Fig. 8). The low-temperature mortality was significantly lower in the dsRNA-treated larvae than in the controls. This result indicated that silencing DarmSDH, DarmTPS and DarmGLK affected the cold-tolerance capacity of the larvae. In addition, the partial silencing of target genes shown by qRT-PCR and the mortality in the low-temperature analyses demonstrated the feasibility of significantly reducing DarmSDH, DarmTPS and DarmGLK gene expressions using dsRNA.

Ideally, dsRNAs would be categorically specific, regulating only the target gene of interest^85^. However, a growing body of evidence demonstrates that this is not necessarily the case. These reports indicate that dsRNAs can affect the expression of unintended targets. Nonetheless, the potential for off-target silencing does not override the enormous potential of RNAi as a tool for the investigation of gene function^86^.

The simplest explanation for these findings is that DarmSDH, DarmTPS and DarmGLK are important for the cold tolerance of the overwintering larvae. Although we were not able to completely silence these three genes, the partial knockdown clearly affected mortality from low temperatures. These RNAi experiments provide evidence *in vivo* that DarmSDH, DarmTPS and DarmGLK are involved during the overwintering stage. The results of this study may serve as a foundation for future studies that aim to elucidate related cold-tolerance, target genes to interfere with insect overwintering behaviour.

## Conclusion

In this paper, we describe certain physiological and biochemical features of cold hardiness in a wild population of *D. armandi* larvae, with special attention to overwintering. Changes in enzyme activities and in mortality at low temperatures were related to the seasonal development of the overwintering stage. We have demonstrated the existence of DarmSDH, DarmTPS and DarmGLK genes in *D. armandi* larvae and characterized these genes. The molecular characterizations of these three genes and the analyses of their expression patterns are first steps to understanding the molecular mechanisms responsible for their potential pest control applications. The functional characterizations of the DarmSDH, DarmTPS and DarmGLK by RNAi demonstrate that these three genes are very important during the overwintering period and for developing a cold-tolerance capacity. Further studies on the mechanisms by which these cold-tolerance genes deliver signals to neurons are needed for a complete understanding of their concerted evolution in insects.

## Materials and Method

### Insect collection

The larvae of Chinese white pine beetles were collected in the Huoditang Experimental Forest Station of Northwest A&F University. The sampling site was located on the southern slope of the middle Qinling Mountains (33°17′–33°27′N, 108°22′–108°40′E), Shaanxi, China.

Overwintering Chinese white pine beetle, *D. armandi* larvae were collected from host trees Chinese white pines, *P. armandi* on the 10^th^ of each month between October 2014 and March 2015 at six occasions from the above site. The larvae were collected from three sample plots in each month, each of which was 20 m × 20 m. We chose 5 trees in each sampling plot according to the five-point sampling method. We peeled off the phloem (20 cm × 20 cm) of each host tree from four directions to collect the larvae. The larvae were then transferred to the laboratory in the dark environment and used to examine the survival rate with exposure to low temperature, measure the physiological parameters, determine the activities of enzymes and extract the DNA.

### Insects and treatments

The larvae of *D. armandi* were collected on the December 10^th^, 2014 and May 10^th^, 2015. The larvae were treated with different temperature (4, 0, −2, −4, −6, −8 and −10 °C) for different times (1, 3, 6, 12, 18 and 24 h). We used 2520 larvae in total for the treatments with dry electrothermostat (BG25, Hangzhou LongGene Scientific Instruments Co., Ltd., Zhejiang, China). After exposure to low temperatures, the specimens were maintained at room temperature for 1 h, before detecting body movements when stimulated with a tweezers^5^,^87^. We chose the larvae which were alive to extract RNA.

### Mensuration of physiological indices

Three physiological indices were measured monthly from October 2014 to March 2015 to quantify the physiological variations in larvae, including the contents of sorbitol, trehalose and glycerol in the bodies of larvae.

Total body glycerol: A whole larva was treated as one sample, and twenty larvae were used for each treatment with 3 replicates. The fresh weight (FW) of an individual larva was measured with an electronic analytical balance (AL204, Mettler Toledo, Switzerland) at nine sampling time points. Twenty larvae were placed into a 2.5 mL Eppendorf tubes and dried at 60 °C for 72 h. The dried larvae were homogenized with 2 mL of 70% ethanol and centrifuged at 3000 r/min for 10 min. The pooled supernatants from three replicates of the process were abandoned, and the rest of the pellet was used to isolate glycogen, according to the method reported by Ohtsu *et al*.^88^. Subsequently, 3 mL of 10% (v/v) trichloroacetic acid was added to the residue and the mixture was boiled in water for 15 min before cooling and centrifugation at 3000 r/min for 15 min^89^.

The supernatant was used to detect the glycerol levels. The glycerol content was determined using the phenol and sulfuric acid method^89^,^90^. The absorbance was determined at 650 nm using a spectrophotometer (UV-1800PC, Shanghai Mapada Instrument Co., Ltd., Shanghai, China), and the results were expressed as mg/g. A calibration curve was obtained by measuring glycerol standards at 11 concentrations ranging from 0 to 5.0 mg/mL in incremental steps of 0.5 mg/mL^90^.

The concentration of trehalose and sorbitol: 0.1 g larvae were treated as one sample, and used for each treatment with 3 replicates. The fresh weight (FW) of an individual larva was measured with an electronic analytical balance (AL204, Mettler Toledo, Switzerland) at nine sampling time points. Anthrone colorimetry method was adopted to determine the concentration of trehalose. This compound have specific absorbing peak at 620 nm after grinding, extraction, quiescence, centrifugation, boiling and cooling. The principle of determining the sorbitol content is that sorbitol can form a blue complex with Cu^2+^ in alkaline solution. These two substances’ content levels were determined with a spectrophotometer (UV-1800PC, Shanghai Mapada Instrument Co., Ltd., Shanghai, China) using the relevant content kit ((SY-6 for trehalose and SY-8 for sorbitol, respectively), Suzhou Comin Biotechnology Co., Ltd., Jiangsu, China) according to the kit protocol.

### Enzyme activity assays

Ten kinds of enzyme activities were determined monthly from October 2014 to March 2015 according to each protocol of the kit (FY-2, FY-1, FY-3, NY-6, AY-4, NY-2, SY-7, SY-9, SY-8 and KW-1 for catalase (CAT), Superoxide dismutase (SOD), peroxidase (POD), Lactate dehydrogenase (LDH), malate dehydrogenase (MDH), Pyruvate kinase (PK), trehalose-6-phosphate synthase (TPS), Sorbitol dehydrogenase (SDH), glycerol kinase (GLK) and Acetylcholin esterase (AchE), respectively. All of the enzyme activity kits were purchased from the Comin Biotechnology Company (Suzhou, Jiangsu, China).

### Mortality at low temperature

Larvae were collected from November and December 2014 and then transferred to a dry electrothermostat (BG25, Hangzhou LongGene Scientific Instruments Co., Ltd., Zhejiang, China) (n = 60 larvae, 3 replicates of 20 larvae, for each temperature and time). Separate larvae were exposed to seven different constant temperatures (4, 0, −2, −4, −6, −8 and −10 °C) for different time (0.5, 1, 1.5, 2, 2.5, 3, 3.5, 4, 4.5, 5, 5.5 and 6 h). Each individual larva was placed in a 200 μL perforated Eppendorf PCR tube and kept dry. After exposure to low temperatures, the specimens were maintained at room temperature for 1 h before the detection of body movements when they were stimulated with tweezers^5^,^87^. The median lower lethal temperature (LT_50_), the temperature that caused 50% mortality was estimated with logistic regression.

### RNA isolation and cDNA synthesis

Total RNA was extracted following the protocol of the RNA extraction kit (UNlQ-10 Column Trizol Total RNA Isolation Kit, Sangon Biotech, Shanghai, China) for all of the above treatments. The quality of total RNA was detected by NanoDrop ND-1000 Spectrophotometer (Nano Drop Products, Wilmington, DE, USA). Finally, 500 ng total RNA (OD260/OD280 = 1.80–2.10) was used for cDNA synthesis. First-strand cDNA was synthesized by using PrimeScriptTM RT reagent Kit with gDNA Eraser (Perfect Real Time) (Takara Biotech, Dalian, China) according to the manufacturer’s instructions.

### Gene cloning and sequence analyses of DarmSDH, DarmTPS and DarmGLK

The synthesized cDNA obtained from the sample was used as a template in PCR reactions. Each pair of specific primer was designed by Primer Premier 5.0 (Premier Biosoft International, Palo Alto, CA, USA) (Table 2), including sorbitol-dehydrogenase (SDH), trehalose-6-phosphate synthase (TPS) and glycerol kinase (GLK). All PCR amplifications were performed with a S1000™ Thermal Cycler (Bio-Rad, Hercules, CA, USA) in a final mixture volume of 50 μL, containing 25 μL 2 × Taq Master Mix (CoWin Biotech, Beijing, China), 0.5 μL of each primer (10 μM, Sangon Biotech, Shanghai, China), 1 μL 1st cDNA template (synthesized using 500 ng antenna total RNA) and 23 μL RNase-free water. The amplification was performed under the following program: an initial denaturation at 95 °C for 3 min, followed by 30 cycles of 95 °C for 30 s, 60 °C for 30 s (SDH); 52 °C for 30 s (TPS); 59 °C for 30 s (GLK) and 72 °C for 30 s, and a final extension at 72 °C for 10 min. The PCR products were visualized on 1% agarose gels after being stained with 1 × DuRed and compared with a 2K plus DNA marker (Beijing TransGen Biotech Co., Ltd., Beijing, China). After that, they were purified using the Gel Purification Kit (Spin-column) (Bio Teke, Beijing, China), connected with the pMD™ 18-T Vector (TaKaRa Bio Inc, Dalian, China), and then transformed into DH5α chemically competent cells of *Escherichia coli*. The transformants (white colonies) were selected on Amp/X-gal/IPTG plates, and a total of 15 clones with inserts were sequenced directly by GenScript USA Inc (Nanjing, China). The obtained partial sequences were manually edited with DNAMAN and blasted against the NCBI database.

### Expression profile analysis of DarmSDH, DarmTPS and DarmGLK

The transcripts of different low temperature, different time and RNAi treated larvae were measured by using a CFX-96 real-time PCR Detection System (Bio-Rad, Hercules, CA, USA) and the Roche SYBR Green system (Roche Diagnostics GmbH, SandhoferStraße, Mannheim, Germany). Actin gene (GenBank accession number: KJ507200) of *D. armandi* was used as endogenous control to normalize the target gene expression. The primers of the target and reference genes were designed by Primer Express 5.0 (Applied Biosystems, Carlsbad, CA) (Table 2). qRT-PCR reactions were conducted in 20 μL reaction mixtures, each containing 10 μL of 2 × SYBR Premix Ex Taq (Roche Diagnostics GmbH, Sandhofer Straße, Mannheim, Germany), 0.3 μL of each primer (10 μM), 1μL of cDNA, and 8.4 μL of sterilized H_2_O. A three-step amplification procedure was used: 95 °C for 10 min and 40 cycles at 95 °C for 15 s, 57 °C (SDH); 60 °C (TPS and GLK) for 30 s and 72 °C for 25 s. Experiments for test samples, endogenous control, and negative control were performed in triplicate to ensure reproducibility. Relative quantification was performed by using the comparative 2^−ΔΔCt^ method^91^. All data were normalized to endogenous actin levels from the same samples.

### RNA interference

According to the manufacturer’s instructions of the T7 Ribo-MAX™ Express RNAi System (Promega, Madison, MI, USA), primers (Table 2) were designed to synthesize the partial region of three genes (glycerol kinase gene −387 bp, trehalose-6-phosphate synthase gene −440 bp, sorbitol dehydrogenase −420 bp, respectively). The final dsRNA products were eluted into DEPC water, stored at −80 °C and used within 1 week.

Before injection, a 1% agarose plate was made and placed on an ice tray. *D. armandi* larvae under 70% ethanol anesthesia were immobilized on the agarose plate with the abdomen directed airward using manual forceps. Afterwards, 0.05 μL DEPC treated water or dsRNA solution (0.1 μM) was injected in each *D. armandi* larvae using a PLI-100 Pico-Injector (Harvard Apparatus, Holliston, MA, USA). Each treatment contains 90 larvae in triplicates. After injection, *D. armandi* larvae were kept in a refrigerator at 4 °C. 15 larvae in triplicate were selected per 24 h, 48 h and 72 h, frozen in liquid nitrogen, and then stored at −80 °C before qRT-PCR analysis (see above). Larvae injected for 24 h, 48 h and 72 h were tested for the mortality, and repeated three times each.

### Statistical analysis

Statistical analyses of overwintering larvae data among trehalose, sorbitol and glycerol content, and enzyme activity assays in *D. armandi* were performed using ANOVA followed by Tukey’s tests for multiple comparisons to detect significant differences. To determine the LT_50_ value of the different exposure time, binary logistic regressions were used to calculate the temperature at which 50% mortality occurred^92^. Relative expression values for all of the genes were determined using the Ct (ΔΔCt) method and analyzed with Microsoft Excel 2003 (v.11.0.5612)^61^. To evaluate significant differences in the expression for each gene, 2^−ΔΔCt^ values were subjected to one-way ANOVA to determine if the gene expression was different among the treatments. The data and figure analyses were performed with SPSS 18.0 (IBM SPSS Statistics, Chicago, IL, USA) using Sigma Plot 12.5 software (Systat Software Inc., San Jose, CA, USA). All of the data were expressed as the mean ± SE, except for the survival at low temperatures.

## Additional Information

**How to cite this article**: Wang, J. *et al*. Cold tolerance and silencing of three cold-tolerance genes of overwintering Chinese white pine larvae. *Sci. Rep.* **6**, 34698; doi: 10.1038/srep34698 (2016).

## Figures and Tables

**Table 1:** Changes of lethal temperature for 50% mortality (LT_50_) in overwintering larvae of *D. armandi* for each exposure time length (0.5, 1, 1.5, 2, 2.5, 3, 3.5, 4, 4.5, 5, 5.5 and 6 h).

Exposure time (h)	LT_50_ (°C)	95% CI (°C)		LT_50_ (°C)	95% CI (°C)	
	Nov. 2014	Lower	Upper	Dec. 2014	Lower	Upper
0.5	−5.7	−5.8	−5.6	−10.4	−10.5	−10.3
1	−5.4	−5.5	−5.3	−9.0	−9.1	−8.9
1.5	−4.8	−4.9	−4.8	−8.0	−8.2	−7.9
2	−4.4	−4.7	−4.2	−8.0	−8.1	−7.9
2.5	−4	−4.1	−3.7	−7.8	−7.9	−7.5
3	−2	−2.1	−1.8	−6.5	−6.7	−6.3
3.5	−0.9	−1.0	−0.8	−5.9	−6.2	−5.7
4	0	−0.2	0.3	−3.7	−3.9	−3.6
4.5	4.4	4.2	4.5	−2	−2.2	−1.9
5	4.7	4.5	4.9	4.1	3.9	4.3
5.5	4.9	4.8	5.1	4.3	4.2	4.5
6	5.4	5.2	5.6	5	4.8	5.1

**Table 2:** Primer sequences and annealing temperature used in the research.

Gene	Direction 5′ → 3′	Sequence	Annealing temperature	Used for
DarmGLK	Forward	CGCCGATTGCTGATTGCCTAA	59 °C	PCR
	Reverse	CCGTTAGAAAGGCGATTAGGG	59 °C	PCR
	Forward	CCTAATCGCCTTTCTA	60 °C	qPCR
	Reverse	CCATTTGCGGTCTTCC	60 °C	qPCR
	Forward	TAATACGACTCACTATAGGGAGGAATGTTCGGGAGG		RNAi
	Reverse	TAATACGACTCACTATAGGGGAGGAAGCGGAAGAGC		RNAi
DarmTPS	Forward	AGGCAGGCTTCAAAGTGGGGA	52 °C	PCR
	Reverse	GTAATAGCGAAGAGGCCCGCACCGA	52 °C	PCR
	Forward	TCACCAACTTCCAACCGA	60 °C	qPCR
	Reverse	CGAGCCTCCAAAGCCATA	60 °C	qPCR
	Forward	TAATACGACTCACTATAGGGCGGGATGGTGCCTGGAT		RNAi
	Reverse	TAATACGACTCACTATAGGGGCGAGGAAGCGGAAGA		RNAi
DarmSDH	Forward	GTTGGGCTGCTCTCCTAATA	60 °C	PCR
	Reverse	GTGGTTGGTATTTGCGGCTC	60 °C	PCR
	Forward	GCAACTTGGTCGCCTG	57 °C	qPCR
	Reverse	GAGCGGGACCTATCGG	57 °C	qPCR
	Forward	TAATACGACTCACTATAGGGCGATTGTTGGGCTGCTCT		RNAi
	Reverse	TAATACGACTCACTATAGGGTGGCGACTACCATCTTTGC		RNAi

**Table 3:** Putative amino acid identity of SDH, TPS and GLK cDNAs isolated from *Dendroctonus armandi* larvae with sequences in insects.

*D. armandi*	BLAST matches in GenBank			Identity
Name	Species	Name	Accession number	
DarmSDH	*Dendroctonus ponderosae*	Hypothetical protein	ERL94043.1	95%
	*Tribolium castaneum*	Sorbitol dehydrogenase	XP_972368.1	72%
	*Camponotus floridanus*	Sorbitol dehydrogenase	EFN71779.1	66%
DarmTPS	*Dendroctonus ponderosae*	Hypothetical protein	ENN76104.1	83%
	*Tribolium castaneum*	Alpha-trehalose-phosphate synthase	XP_975776.2	51%
	*Drosophila albomicans*	Trehalose-6-phosphate synthase	BAR90544.1	49%
DarmGLK	*Dendroctonus ponderosae*	Hypothetical protein	ERL93318.1	95%
	*Tribolium castaneum*	Glycerol kinase	XP_008190639.1	77%
	*Papilio machaon*	Glycerol kinase	KPJ09152.1	70%

**Table 4:** F value and Significance after dsRNA-injected for 24 h, 48 h and 72 h.

Gene Name	Time for dsRNA injection	F Value	df	P-value	Significance
DarmSDH	24 h	4910.122	2	<0.001	**
	48 h	2660.157	2	<0.001	**
	72 h	172.591	2	<0.001	**
DarmTPS	24 h	16399.752	2	<0.001	**
	48 h	15609.652	2	<0.001	**
	72 h	309.275	2	<0.001	**
DarmGLK	24 h	16062.987	2	<0.001	**
	48 h	152.835	2	<0.001	**
	72 h	54.340	2	<0.001	**
